# Exploring human behavior change in equine welfare: Insights from a COM-B analysis of the UK's equine obesity epidemic

**DOI:** 10.3389/fvets.2022.961537

**Published:** 2022-11-08

**Authors:** Tamzin Furtado, Elizabeth Perkins, Gina Pinchbeck, Catherine McGowan, Francine Watkins, Rob Christley

**Affiliations:** ^1^Institute of Infection, Veterinary and Ecological Sciences, University of Liverpool, Liverpool, United Kingdom; ^2^Institute of Population Health Sciences, University of Liverpool, Liverpool, United Kingdom; ^3^Dogs Trust, London, United Kingdom

**Keywords:** qualitative research, behavior change, equine welfare, overweight and obesity, COM-B behavior change wheel

## Abstract

While equine obesity is understood by equine professionals to be a serious and widespread welfare problem, thus far approaches to reducing the prevalence of obesity in the UK's leisure horses have mainly been limited to educating owners about the dangers of obesity in their horses. In human health, approaches to behavior change encourage holistic thinking around human behavior, recognizing the importance of the connection between the individuals' knowledge, attitudes, habits, and the social and physical environments. This study used qualitative data from interviews with horse owners and professionals, open-access discussion fora and focus groups in order to collate extensive information about the factors shaping the UK's equine obesity crisis. The data were initially analyzed using a grounded theory method to determine the common themes, and were then analyzed using the COM-B model of behavior change, in order to identify areas where human behavior change might be better supported. The analysis highlighted the importance of a holistic approach to behavior change, since all areas of the COM-B were important in limiting owners' recognition of, and response to, equine obesity. For example, environments and social norms limited the likelihood of owners proactively managing horse weight, and owners also found it difficult to identify overweight horses, and evaluate the risks of long-term health issues as a result of weight, with short-term negative impacts of weight management. While interventions often aim to educate owners into changing their behavior, this analysis highlights the importance of creative and holistic approaches which work alongside the owners' motivations, while shaping the social and physical environments.

## Introduction

Equine obesity is a multifaceted welfare problem for UK horses, made more complex by the varying recognition it receives from different groups of stakeholders. Obesity is considered by equine professionals to be a serious and preventable welfare problem with potentially life-threatening consequences for horses caused by obesity-related diseases such as Equine Metabolic Syndrome (EMS), laminitis, and osteoarthritis (1, 2). Yet, several studies have shown that owners fail to recognize horses who are overweight (3–7) furthermore, even when obesity is recognized, equine professionals report that they do not think owners make enough changes to decrease weight (8).

Most existing efforts to reduce obesity in horses have been aimed at educating owners about the dangers of obesity (1, 9, 10), and many of those working in animal welfare or veterinary sciences subscribe to the view that education will assist in reducing the obesity prevalence (3). This view of behavior change follows a neoliberal discourse, and is common in the biomedical model of public health (11). In this model, each individual is considered an agent in their health choices; someone who has the ability to make and act upon responsible decisions to optimize their health. In this context, owners of overweight/obese horses are seen as agents who are making poor choices for their animals, and hence education about the potential harm to their animal's health could help to inform and alter their behavior. However, this idea of health agency is now contested in human public health due to an improved understanding of the factors which contribute to behavior change, including the role of our emotions and the social and physical environments (12). As a result, thought needs to be given to how animal owners can be encouraged to manage the health of their animal using strategies that move beyond education alone.

Current understanding of public health and behavior change suggests that awareness of a problem does not necessarily alter behavior (11, 13). As the science of behavior change has evolved, a growing body of researchers have argued that knowledge, and even intention, are not necessarily linked to actual behavior; instead, intention is theorized to relate to only 20% of our actions (14), with attitudes *following* behavior rather than the other way round. The key to encouraging behavior change is to first establish small changes in people's behavior, which will then lead to a change in their attitude and motivation (15). This theory was used successfully with the *Stoptober* Campaign (16) for smoking cessation; unlike many previous public health campaigns, *Stoptober* did not focus on education and the reasons for stopping smoking, but instead encouraged individuals to make a public commitment to changing their behavior for a set time period—one month—by making a pledge on social media. The *Stoptober* campaign was highly effective in increasing quit-attempts, with a 50% increase in attempts compared to other months (16).

Other behavior change programmes have followed suit by focusing on assisting people to change their behavior in a positive and socially recognized way, rather than focusing on educating them about the “negative” behavior that they are already doing. These approaches are supported by researchers in behavioral science. In a review of the use of cognitive behavioral therapies (CBT) for weight management, researchers determined that altering behavior and habits was the most influential way of reducing levels of human obesity (17). It may be the case that aspects of such behavioral therapies could be utilized to encourage horse owners to change their feeding behaviors, for example by encouraging the owner to replace treat feeding with attention or grooming (18).

Many models of behavior change have been developed to help to explore the links between motivation, intention, and behavior. The central aspects of a number of these models have been combined to produce the COM-B model of behavior change (19) (COM-B: Capability-Opportunity-Motivation—Behavior. In this model, each of the three components (Capability, Opportunity and Motivation) are subdivided into two further areas. A person's motivation includes their conscious understanding of the issue (*reflective* motivation) and unconscious biases, emotions and habits (*automatic* motivation). Behavior may also be affected by their capability to perform the behavior (in relation to both their *physical* and *psychological* capacity), as well the opportunity to perform the behaviors in the real world, both *physically* and *socially*.

The COM-B model is well-recognized and validated (20), and has been used in multiple human health settings (21–25). Its use is now being extended into human behaviors which affect animal health (26, 27). It provides a useful framework for considering the requirements which are important when altering a specific behavior.

Behavior change science within animal welfare is an evolving field of study. Understanding the factors involved in changing human behaviors in relation to animal keeping is important in order to be able to implement change for improved animal welfare. For example, many educational programmes related to companion animal obesity have focused primarily on warnings about the health risks involved in obesity, with the blame laid at the feet of the veterinarians for not communicating the severity of the long term risks and/or owners for not responding effectively (28–30). However, encouraging environmental, social, or habit-changing behavioral interventions could potentially result in more lasting change.

Use of the COM-B model provokes a wider consideration of the factors which may be impacting on horse owner behavior in relation to obesity in horses. For example access to grass, an environmental factor (part of *physical opportunity* in the COM-B) is a major risk factor for obesity (31), particularly if the pasture was originally intended for dairy cattle, whose energy requirements exceed those of horses because of the energy requirements of producing milk (32). However, the COM-B has not previously been used in relation to obesity in horses, though its use could provide important insights. This study uses qualitative data to explore the experiences, perceptions and motivations of horse owners, subsequently analyzing the data with the COM-B in order to illuminate areas where change could facilitate better equine weight management.

## Methods

This study utilized qualitative data collected over a three-year period, including semi-structured interviews with 28 horse owners and 19 equine professionals (e.g., veterinarians, farriers, nutritionists and a behaviorist), 17 discussion threads about equine obesity and its management from open-access UK discussion fora, focus groups with 21 horse owners interested in managing weight, and extensive field notes. A detailed description of the methodology is provided elsewhere (7, 33). Briefly; horse owner participants (including owners of obese and healthy-weight horses) were recruited to a study about horse health and wellbeing, which included but was not limited to weight management. Interviews were primarily conducted in-person at the horse's yard, between August 2017 and July 2019; five interviews were conducted *via* phone for convenience, but accompanied with photographs or a video-tour of the yard. Two focus groups were also held in-person, specifically around the challenges faced by horse owners in managing equine weight. Interviews with equine professionals aimed to discuss the professionals' experiences of assisting owners in managing weight. The study was approved by the University of Liverpool Veterinary Ethics committee (approval number 457), and all participants in the interviews and focus groups provided informed consent prior to taking part. Participants are briefly outlined in Table 1.

**Table 1:** Horse owning and equine professional participants in the interview phase of the project.

**Participant number**	**Participant type**	**Horse description**	**Interview conducted:**
	Horse owner	TB	Face to face
1.	Horse owner/livery manager	Welsh cob, Welsh Sec A and Andalusian	Face to face
2.	Horse owner	Arab x Welsh	Face to face
3.	Horse owner	Irish sport horse	Face to face
4.	Horse owner	Thoroughbred	Face to face
5.	Horse owner	Lightweight cob	Face to face
6.	Horse owner	Highland mare	Face to face
7.	Horse owner	Mid weight cob	Face to face
8.	Horse owner	Irish draft	Face to face
9.	Horse owner	Irish sport horse	Face to face
10.	Horse owner	Welsh sec D	Face to face
11.	Horse owner	3x shetlands, Irish sports horse, cob, gypsy cob	Face to face
12.	Horse owner	2x Cleveland bay	Phone
13.	Horse owner	Gypsy cob	Face to face
14.	Horse owner	Thoroughbred	Phone
15.	Horse owner	Cob x and Welsh x	Phone
17.	Horse owner	Gypsy cob	Face to face
17.	Horse owner	Dutch warmblood and Connemara	Phone
18.	Horse owner	Cob, Gypsy cob	Face to face
19.	Horse owner		Face to face
20.	Horse owner	Irish sport horse and Connemara	Phone
21.	Horse owner	Gypsy cob, Irish draft x Clydesdale, Thoroughbred X	Face to face
22.	Horse owner	Highland	Face to face
23.	Horse owner	Dales, cob	Face to face
24.	Horse owner	Thoroughbred	Face to face
25.	Horse owner	Irish sport horse and welsh	Face to face
26.	Horse owner	Morgan	Phone
27.	Horse owner	Welsh sec D	Phone
28.	Yard manager		Face to face
29.	Yard manager		Face to face
30.	Farrier		Face to face
31.	Nutritionist (independent)		Face to face
32.	Farrier		Face to face
33.	Farrier		Face to face
34.	Equine welfare staff—charity		Face to face
35.	Behaviorist		Face to face
36.	Yard manager (farmer)		Face to face
37.	Farrier		Face to face
38.	First opinion veterinarian		Face to face
39.	First opinion veterinarian		Phone
40.	First opinion veterinarian		Phone
41.	First opinion veterinarian		Phone
42.	Nutritionist		Phone—(collected as part of relevant past project)
43.	Nutritionist		Phone—(collected as part of relevant past project)
44.	Nutritionist		Phone—(collected as part of relevant past project)
45.	Nutritionist		Phone—(collected as part of relevant past project)
46.	Nutritionist		Phone—(collected as part of relevant past project)

The data were audio-recorded, transcribed verbatim, then fully anonymized by altering any names, places, or other identifiable details. Once transcriptions had been checked against the original audio-recording, the data were analyzed in a qualitative research software programme (NVivo 10) using a grounded theory approach based on the work of Charmaz (34). Grounded theory is a well-recognized methodology for conducting qualitative research studies, and relates to a process of analyzing text by “grounding” in the data (34, 35); the researcher suspends their own pre-existing knowledge and opinions about the topic under study, instead immersing themselves closely in the experiences that the participants have shared *via* the data. Initially, transcripts were read closely, and initial impressions about items of importance marked. This was followed by several rounds of increasingly detailed “coding” to create categories of meaning or “themes” (for example, they might code areas in the text which are about the descriptions of overweight equine bodies under a category initial called “perceptions of overweight”). As more texts and transcripts were added to the data, a more holistic picture was built of the experiences of the participants; at this point, the initial categories were refined, altered, or combined, and over-arching themes explored. Hence, the resulting set of themes and sub-categories provide an analytical reflection of the experiences of the participants included in the study.

Once a full understanding of horse owners' experiences during equine weight management was mapped out, the COM-B model was then used to further reflect on the data. Each section of the COM-B model was considered in turn, with the aim of determining which themes in the data (if any) matched the section of the COM-B model. For example, under the section “physical opportunity”, themes which related to the impact of the physical environment around the horse and owner were collected. This analysis highlighted areas where horse owners' behavior around weight management might be influenced in order to facilitate change.

## Results

Here, each of the COM-B model's sections is examined turn, first explaining the meaning of that section, and then describing how that section relates to the data related to equine obesity gathered during this project.

Table 2 provides an overview of the types of findings for each section of the COM-B model.

**Table 2:** Application of COM-B components to two relevant aspects in equine obesity: Recognition of equine overweight, and management of equine weight.

**Component of COM-B**	**Sub-component of COM-B**	**Recognition of equine weight**	**Management of equine weight**
Capability	Psychological	The ability to recognize that the horse is overweight was often compromised. Owners often knew of the existence of body condition scoring scales, but rarely knew how to apply these to their horses' bodies; instead they simply “eyeballed” them for an overall picture of condition, which led them to focus on areas such as the abdomen, which are not good markers of fat absence or presence.	Lack of knowledge of how to apply weight management strategies, and adapt according to individual situation
	Physical	N/A	Inability to physically manage heavy procedures (e.g., soak hay) Physical ability of the horses to be exercised sometimes lacking (e.g., if retired, injured)
Opportunity	Social	Social norms promote fat as part of healthy equine bodies (e.g., in magazines, and in some show classes)	Social norms promote the idea that weight management is usually “cruel” or “mean” because it is depriving the horse; language used supports this e.g., “starvation paddock” and “work” for exercise.
	Physical	N/A	Frequent lack of physical means to manage weight; e.g., many livery yards do not have access to grass free/low grass turnout areas, disallow electric fencing, etc. Lack of locations to safely exercise the horse (for example, poor bridleway infrastructure and busy roads)
Motivation	Automatic	Familiarity with horse (from seeing it daily) led to many owners being unable to objectively determine increases in weight. Internally held views about how some breeds (e.g., cobs, native ponies) were “meant” to look led owners to a lack of understanding about which areas were body fat and which areas were simply the horses' shape	Habitual behaviors and care practices such as feeding and exercises behaviors were hard to change; horses reinforced those habitual behaviors by positively reinforcing behaviors they liked (e.g., “nickering” at owners for feed) and by avoiding situations they disliked (e.g., jumping out of a low-grass paddock into one with more grass; becoming grumpy; refusing to exercise). Many owners described fear of activities such as riding or hacking, which would help enable them to exercise their horse. This was particularly evident in relation to fear of hacking out on roads
	Reflective	Compromised ability to apply their knowledge of health risks from obesity in the abstract, to their individual animal	Short term welfare often takes priority when weighing up the perceived negative impact on the horse of the weight management strategies themselves (e.g., feeding less) versus the harmful potential effect of fat long-term. The prioritization of short-term welfare was reinforced by the behavior of horses when those weight management strategies limited them (e.g., if horses became grumpy due to his/her management, owners were likely to dislike this management option).

### Capability: Psychological

This aspect of the model explores whether individuals have the knowledge to perform the required behavior, and have the cognitive skills to carry the behavior out in practice. This category was important in two areas: firstly identifying whether the horse is overweight and why that matters (e.g., understanding and assessing their horse's body fat and the need to make a change in order to improve health and wellbeing, and understanding the links between obesity and other health problems), and secondly, understanding how to make changes to improve horse health (e.g., the knowledge of how weight management strategies might impact weight and how to apply these strategies flexibly over time).

Our data indicated that owners lacked the knowledge to effectively identify excess fat from their horses (7), particularly if the horse was a heavier breed such as a native-breed pony (e.g., a Shetland or Welsh pony) or cob. Importantly, this issue seemed to be related particularly to *their specific horse*, rather than knowing how to identify fat on horses in general. For example, P16 is a professional groom at a horse charity, and has had training in condition scoring. Nevertheless, she needed her veterinarian's help to identify fat on her cob pony:

P16: *Sometimes when you see a horse every day, you don't really appreciate how fat it is. He lost some weight when I first got him, over the winter. I thought we were doing alright. But then the vet came out to see him and he was like, “He's still really fat, you know?” I was really disappointed*.

Other respondents showed similar knowledge of the existence of condition scoring charts, yet difficulty in knowing when to apply changes to the horse's management. For example, P23 owned a large gypsy cob mare, and displayed her knowledge of condition scoring, placing her own horse as very fat/nearing obese (a point she repeatedly mentioned in the interview, pointing out fat pads throughout and saying to the horse “*you're fat! Look how fat she is!*”) whilst simultaneously suggesting she did not need to make changes to the horse's management:

*P23: She's not ridiculously obese for her sort of cob but she's not slim at the moment. Here is definitely fat “[indicates shoulder fat pad]…. If I think they're getting obese then I'll start doing something about it.”*

These examples highlight how owners often had some knowledge of how and where fat might be located on a horse, but applying their knowledge to their horse's body, then subsequently making changes to management, proved problematic.

Most owners had an understanding that excess body fat was a health risk to their horse, and they often related this particularly to laminitis. However, because owners found it hard to identify how much fat was on their specific animal, most owners in this study had not made changes to the horse's management to reduce weight, until an acute health issue such as laminitis occurred, requiring immediate action.

The results highlight that education alone is unlikely to solve equine obesity: owners showed that they may already be aware of the issues caused by weight, and where weight might sit on a horse—but the issue in the psychological capability section of the COM-B model which limits them actually acting, is knowing at what point on their specific horse fat becomes excessive and requiring intervention. Of course, the psychological capability could also be impacted by other areas of the COM-B model, including the person's motivation to make a change.

While owners in this study understood that a range of weight management strategies were available, they also found it hard to identify what would work within the environment in which they kept their horse, while also maintaining other aspects of their horse's physical and mental health, protecting the land, as well as being manageable within the owners' resources.

Designing weight management to meet these varied needs therefore requires knowledge of a range of different available strategies, which was sometimes problematic. Respondents and forum participants alike commonly focussed on lunging, riding, soaking hay, strip grazing, and using muzzles in order to manage weight. With only a limited range of options, owners could therefore quickly feel that they had “tried everything”, particularly given the fact that horses could easily thwart efforts to manage weight by, for example, learning to remove their muzzles or breaking fencing. For example, P21a described her feelings of futility: “*you hit a point and you're like, I don't know what else I can do here*”, having tried multiple different weight management methods for her cob mare, and had issues with each (the mare became aggressive when hungry, would not lunge, was too broad to wear a saddle).

Across the dataset, forty different strategies were identified by owners (36), incorporating a range of relatively unusual practices which owners found fit more easily within their lives. For instance, these included riding one horse and leading another; using paddock configurations designed to make the horse travel the maximum distance to reach its resources; borrowing sheep to eat the grass; turning the horse out with youngsters to encourage play; and putting in a sand-turnout area. Interestingly, these ideas were relatively unfamiliar for other owners, highlighting a lack of knowledge of the more unusual weight management strategies.

As well as knowledge of strategies, owners also needed the imagination and flexibility to adapt methods over time according to the horse's health and wellbeing, and owners' capabilities. This often led to weight management being a compromise between what would work for the individual horse and rider:

P36 (qualified behaviorist): *I think different horses respond to different things, some cope with some things and some don't….It almost seems, or I think it seems like you just can't get everything right for each horse. It always seems to be a compromise*.

The psychological capability section of the COM-B model highlights how owners have *some* of the knowledge they need in order to identify overweight animals of strategies to manage overweight animals, but applying that knowledge to everyday life was frequently problematic; sharing unusual or adaptable ideas for weight management was therefore an important gap to fill.

### Capability: Physical

This facet of the COM-B model considers whether the subject has the physical skills and ability (e.g., strength, coordination or dexterity) to perform the behavior in question.

In relation to equine weight management, the need for strength and physical capacity was frequently mentioned due to the need to find new ways of managing horse care, as well as the need to exercise the horse enough to lose weight.

Many older owners in particular discussed their difficulties with heavy tasks such as lifting soaked hay or pushing full wheelbarrows. This limited the weight management options available to them, unless they had assistance from someone else, or could create a new strategy (e.g., some yards have a pulley system to pull heavy haynets from water).

P19: *I just got so tired of soaking hay. It's such hard work. Of course, in the winter, it's awful if it's frozen, because you then can't do it*.

Several owners also spoke about lacking the physical capacity to exercise their horse effectively; for example, one respondent suffered from rheumatoid arthritis and had made the decision to no longer hack her horse due to safety concerns. Similarly, P23 could no longer manage longer or faster rides:

*P23: it's too much. I get a sore back and my knees go and then I can't get off, which is what happened on Sunday of course. I got off too soon*.

Others described previous injuries which still limited them (for example, falls which had broken bones including the vertebrae) and therefore limited the activities they now took part in with their horses. In order to manage weight and allow the horse to continue to exercise, these owners sometimes found other people to ride their horses and perform the tasks which they could not.

An additional physical capability restriction is present in the body of the horse; physical restriction of the horse due to old age or chronic illness was an often-cited reason for the horse not being exercised:

P11: *now she's getting older I think well I don't really want to like start pushing her and everything anymore—I know she's only 20 and you see a lot of horses doing a lot more at 20 but I kind of just thought well I'll give her this time—I won't really push her that much anymore, I'll just do a bit of schooling and occasional hacks*.

Equine bodies were seen as having a further physical limitation in terms of food restriction; as grazing animals, horses were seen as being physiologically unsuited to dieting, and many owners were concerned that dieting their horses would leave them stressed and potentially open to Equine Gastric Ulcer Syndrome (EGUS). Thus, owners described needing to find weight management strategies which did not leave their horses hungry:

P2: *I don't believe in starving horses, because of the stress and the ulcers and I think that's just triggering laminitis*.

Physical capability therefore had a clear impact on both the human and equine sides of the partnership in relation to weight management.

### Opportunity: Social

The social opportunity to perform the desired behaviors relates to the social environment and social norms surrounding an individual, and the consideration of how these might shape behavior. Our data showed that social opportunity plays a major role in horse owners' perceptions of healthy body condition in horses. For example, many participants commented on the social acceptability of overweight horses, in comparison to the lack of acceptability of horses that are even slightly underweight:

P33 farrier*: No one wants to be seen as the one with the skinny horse or the underweight horse, the one who's not looking after it and then they start feeding more*.

P19*: It really upsets me quite a lot that people hold their hands up in horror when they see a thin horse, but if they see a fat one, they don't perceive that to be as much of a welfare issue*.

Horse owners, forum users and professionals alike considered that the social acceptability of obesity in horses was caused by the prevalence of overweight horses in the local community, in the show ring, and in the media.

Social norms center on providing comfort to the horse in ways which may also have obesogenic effects; for example feeds fed as “meals” (an anthropocentric way of feeding, given that horses naturally eat little but often), rugs, and limited but comfortable environments constructed as “bedrooms”:

P17: *I would probably say I'm an over rugger. I'm not going to lie, I probably am. I perceive it as being cold, so I do think I put a rug on him*

P32 (behaviorist): *owners feel that they've got to give their horse something. Completely inappropriate feeding…their pony could really just be on hay or soaked hay. They feel like they've got to feed a balancer and garlic*.

However, weight management strategies were not perceived as a social norm, and were generally constructed as “cruel”:

P45 (Nutritionist)*: They may think it's cruel to reduce the feed and hay down if there is weight to lose or to use a muzzle*.

*P16: We have had a bit of an issue. We had a third horse sharing this field. The other person thought we were starving them all and being very, very cruel*. [in relation to strip grazing in a shared livery paddock]

Social norms were seen by participants as being supportive of potentially obesogenic behaviors, such as feeding and rugging, meaning that owners who were attempting to reduce weight had to be willing to challenge the status quo.

There were also some instances in the data of social opportunity helping owners to better manage their horse's weight: on two of the yards participating in the study, people had created weight management “clubs” which encouraged active weight monitoring and management as a group activity. Similarly, some owners found supportive communities online, where they could share experiences and ideas with other owners who were experiencing similar problems:

P16: *I've found there's a EMS group that's really, really useful with people having ideas, if your horse is only allowed six kilos of hay a day, how to make sure that it lasts as long as possible*.

Such communities normalized weight management and supported owners in finding practical means for overcoming common issues, such as how to manage feeding as restricted diet.

### Opportunity: Physical

Physical opportunity relates to the environmental factors which might limit, or encourage, the individual to perform a behavior. This category was extremely important in considering weight management strategies, with many environmental restrictions identified across the data. For example, owners on livery yards had little control over the type of field their horses were turned out into, and the hours of turnout. Frequently, livery yard managers also disallowed electric fencing, which owners might otherwise have used to modify or reduce paddock size:

P44 (Nutritionist): *grazing management may be difficult, especially if they're on livery yards. Again it's quite disappointing at times that you'll hear, “I'm not allowed to do that,” or, “I can't turn my horse out with a muzzle,” or, “I can't strip some grazing.”*

Given that the time at grass was often considered to be one of the major contributors to equine weight gain, the lack of control over grazing represented a lack of physical opportunity for weight management for many respondents. Further, the *type* of grass available was also considered by many to limit physical opportunity to manage weight, because of the prevalence of high-production rye grasses on many yards, which created an inappropriate environment for horses.

P47 (Nutritionist): *If I had it within my power to destroy off the planet forever rye grass, I would…an awful lot of livery yards … The horses are grazing on former dairy grass. They're high production rye grasses that need fertiliser every year to keep them going. I think they're a major contribution*.

The type of grass coupled with lack of control over grazing management was therefore a major contributor to the lack of physical opportunity for weight management.

Some owners specifically aimed to create physical environments which would better suit horses who were prone to becoming overweight; for example, creating non-grass turnout areas, or extensive “track systems”, whereby horses were turned out in a series of paths which aim to encourage movement and minimize grass access; others re-seeded their fields with grasses that they considered more suitable for horses than the traditional rye grasses.

An additional issue related to a lack of safe spaces to exercise the horse; it was clear across the data that exercising a horse within a confined space was considered “safe” in comparison with hacking on bridleways (where horses may spook or bolt) or roads (which carried additional risks from other road users):

P13: *this lorry driver taunted us, he really was, he was an absolute pig of a man, he used to get so close to us*.

P26: *He* [horse] *would just spin and rear, it could pirouette better than a blinking ballet dancer. Taught me a lot about riding that. That kind of thing. If it's a horse or if there's a loud machine going, he might be a bit cautious of it, or a bin lorry*

Owners therefore described a lack of areas which they perceived would be safe for exercising their horses, which contributed to an overall lack of fitness and therefore increased the likelihood of weight gain.

### Motivation: Automatic

Automatic motivation refers to the “automatic” emotions and behaviors (i.e. those which are not planned but occur spontaneously) which might encourage or inhibit the occurrence of a behavior. This includes emotions about the behavior or factors surrounding the behavior, as well as habits and ritualistic behaviors, which are of extreme importance in everyday activities (37–39). For horse owners, habits and ritualistic behaviors were commonly seen around caring activities, which were perceived as being an important part of routine for both horse and owner. Feeding was a central part of these ritualistic behaviors, and was positively reinforced by horses' responses:

P27 (farrier): *A horse will stand still if it's getting a treat. It's like the owner is just a walking cookie jar almost. They know if they stand still they get a treat or a carrot or something*.

This effect could be pervasive enough that owners and horses co-created rituals around feeding, which owners felt unable to change:

P32 (behaviorist): *she kept him at home, she said that it had got to the point where she couldn't walk out of the house because if she did, he'd be at the gate because he'd be waiting for his custard creams. So, she was restricted to how much she would go outside the house because she felt so guilty*.

Habits and automated behaviors were often problematic, therefore, in promoting obesogenic environments and behaviors.

Some owners were able to incorporate automatic activities into their weight management regime, for example by measuring their horse's weight daily:

P7: *I measure her girth and round here [indicates belly area] on an almost daily basis*.

For this owner, weight monitoring was a habitual part of how she thought about horse care. However, this was quite rare, and few other automated behaviors were associated with weight management strategies.

The emotions of owners around their horses' body condition, care, feeding, and exercise were also important in relation to potentially obesogenic effects. Firstly, it was apparent across the data that underweight body condition was perceived to be a threat to welfare, while the presence of fat was often treated with humor: data collected in field notes included several memes making fun of fat horses (none were found making fun of thin animals), and the language around fat was generally either humorous or cute, such as describing horses as being “*like a hippo*” or “*a chunky boy*”. Such descriptions and responses to fat show deeply held emotions around body image, and are likely to minimize the perceived health threats from fat.

Further, some level of fat was perceived as being indicative of health, and therefore desirable:

P7: *I don't like her too thin, I do like her to have a little bit on her, but at the minute I'm happy with her weight*.

Many owners described the sense of emotional satisfaction that they gained from caring for their horses, maintaining health and restoring health following illness. As such, maintaining fat as a symbol of health could lead owners to feel that they were being “good” owners, leading to satisfaction and fulfillment. Feed could form an important part of this care, not only nourishing the horse's bodies but also providing a positive part of horse-human interactions:

P4: [horse receives] *a handful of something in a bucket after he's worked, so he doesn't come in and think oh work work work. It's something after he's worked to kind of make his day a little bit*

The role of owners' emotions around feeding was perceived by some nutritionists, farriers and behaviorists to be exploited by feed companies, who encouraged owners to feed “meals” causing horses to be excited about food, thus positively reinforcing both owner and horse into repeatedly feeding potentially unnecessary bucket-feed. Contrastingly, as is also shown in the previous quote, exercise such as riding was viewed as negative for horses and a “necessary evil”; this is reflected linguistically with exercise described as “work” and horses “earning their keep” or “earning a retirement”. While this attitude to exercise was implicit, rather than explicit, in the narratives, it is likely that it could contribute to owner negativity over increased exercise as a weight management option. Additionally, many owners described feeling fearful of exercising their horses, particularly in relation to hacking—especially when hacking entailed using roads. Owner fear was often related to specific accidents, injuries, or loss of control, and contributed to low motivation to exercise their horses.

### Motivation: Reflective

Reflective motivation refers to enthusiasm and drive to perform an action as a result of being able to reflect on the reasons for performing it. For example, owners who understand that their horse will be healthier if it is an appropriate weight, and have a clear plan about how to achieve that weight, may be motivated to make a change.

Although most owners described an awareness of the risks of laminitis as a result of excess weight, it was unusual for them to feel motivated to make a change prior to laminitis occurring (possibly because of the aforementioned confusion/lack of psychological capability to identify excess weight). For many owners, reflective motivation strengthened significantly once the horse experienced a weight-related illness, such as laminitis; after this point, the owner was often intensely motivated to monitor and manage its weight.

Interviewer: *When did you first start monitoring her weight?*

P24: *When she got laminitis*

Once laminitis occurred, owners became invested in managing weight in order to avoid a recurrence. Interestingly, other weight-related health conditions (such as osteoarthritis) featured little in discussions about motivation for weight management.

Horse owners also experienced a problem in applying weight management strategies due to competing priorities, which form part of their reflective motivation. Caring behaviors and a positive horse-human relationship were central to owners' perceptions of being a “good” or responsible horse owner, and therefore weight management strategies were viewed as inherently unpleasant for both horse and owner. In order to successfully manage weight, owners therefore needed to prioritize the long-term gains of weight management, over the short-term reduction in equine quality of life which they associated with weight management methods. Horse behavior could significantly influence this process; for example horses who became visibly distressed (for example, grumpy or depressed) or flouted weight management options (jumping out of paddocks; refusing to be caught) were likely to cause owners to rethink their weight management options.

Weight management options which were considered to enhance, rather than restrict, equine welfare were therefore considered preferable by many owners. Track systems were one example which was constructed as an enhanced and enriched environment for horses, despite it also encouraging more movement and less eating. Other owners created non-grass turnout with enrichment (e.g., scratch pads, enrichment hay feeders) in order to provide areas where their horses could live comfortably in small groups. These systems provided an opportunity for positive welfare for horses, which in turn seemed to help owners' reflective motivation with weight management because they did not feel that their horses' wellbeing was reduced.

## Discussion

This study investigated the different human behavioral factors which influence the prevalence of equine obesity, and which may inhibit change. The COM-B model has been used as a framework for analysis, enabling a holistic examination of the influences on behavior.

To our knowledge, the application of the COM-B model to examine human behavior within the horse-human relationship has not previously been described, though it has been used with farm animal welfare issues (40). This study applied the COM-B to data which had first been analyzed according to Grounded Theory principles, without reference to any framework or preconceived ideas. Subsequently, we found that the COM-B model provided a useful means of exploring these themes in the context of behavior change, and that this methodology yielded comprehensive insights into the factors which shaped horse owner behavior around equine weight issues.

The use of the COM-B model highlights the multiple themes within each of the COM-B's six sections which make it difficult for owners to recognize equine weight issues, and to change their management of the animal. Coupled with the obesogenic equine environment in the UK and social norms of equine obesity (33), this produces a potent setting for horse-keeping. The analysis indicates the importance of equine weight management interventions being targeted to a range of different areas of behavioral influence; for example there is no point increasing knowledge and raising awareness (reflective motivation, psychological capability) if owners are effectively inhibited from making changes due to environmental factors (such as the inability to change turnout area on a livery yard, the lack of safe spaces for exercising the horse, or the lack of availability of low-calorie forage) and effect of social norms (such as peer pressure) (41, 42).

While equine obesity has been infrequently studied from a social science perspective, other fields such as human and companion animal obesity have received more research attention, and can provide useful comparisons. For example, in human obesity it is well-established that behavior around dieting and increased exercise is strongly affected by a range of factors, including social influence, motivation, and the physical environment (43–45). This has led to the development of behavior change interventions which move away from increasing knowledge about dieting, to facilitating other factors such as social support and improved self-efficacy in weight management (46). Two major meta-analyses of obesity-related behavioral interventions found that the promotion of “self-monitoring” behaviors (for example, keeping a food diary or regularly recording weight) in interventions were particularly effective (46, 47).

As with horse health, in canine veterinary medicine veterinarians are tasked with encouraging dog owners to recognize and respond to canine overweight, and studies have shown high levels of veterinary frustration over the pervasiveness of this issue (48). Although dog owners consider veterinarians to be their main port of call for dog weight management, veterinarians and owners addressed excess canine weight in differing ways, with veterinarians focusing on simple nutritional reduction and owners preferring diet alteration (e.g., a food brand change) or lifestyle alterations (29). As a result, experts in canine obesity recommend tailoring weight management strategies to the individual dog-owner combination, and focusing on small gains (such as the dog being happier) rather than a specific target weight (49). A meta-analyses of 14 owner-directed interventions to assist in the management of canine obesity found an overall moderate effect of interventions. The interventions included goal-setting, increasing knowledge, and self-monitoring, among others; however, the effect size was relatively homogenous across these interventions, suggesting that no one type of intervention was more successful than any other, which may reflect the diverse strategies needed for weight management. Notably, a failure to reach or maintain target weights in canine health is considered very common (49).

Given that this study reveals barriers to managing equine weight loss which are common to human health, companion animal and equine professionals could, then, follow recommendations from human health studies, particularly focusing on goal-setting and monitoring behaviors. However, the present study has also shown the multiple ways that managing animal obesity has additional layers of complexity. These include the fact that horses are grazing animals and owners consider it important to provide almost constant access to forage in order to prevent gastric complaints. Horses are often kept at livery yards, where aspects of their care may be prescribed by someone other than the owner; horse behavior may either promote or inhibit any changes made by the owners; for example the data showed examples of horses influencing the type of weight management to which they were subject by jumping out of fields, or becoming distressed or aggressive when food is restricted. These factors altered the types of weight management strategy available to horse owners (50), who understandably wanted their horses to experience positive mental affect while their weight was reduced.

Unlike humans and dogs, horses suffer from laminitis: an acute, painful, and sometimes life-threatening result of the metabolic complications of obesity (51). Despite this study's finding that owners were well aware of the links between excess weight and laminitis, they were rarely proactive about weight management. For example, many respondents only began weight management once the horse had already become laminitic; following the horse's recuperation, owners became invested in and vigilant around weight management. This reflects the findings of other aspects of equine management in which owners do not consider their individual horses to be at risk, such as the risk of resistance to anthelmintic drugs (52).

Around 60% of the UK's horses are kept at livery yards (53). This provides an additional opportunity for change at a community level. Some livery yards are now providing highly enriched environments which are specifically targeted at managing overweight or metabolically compromised horses, for example by using non-grass turnout areas with low calorie forage, alongside opportunities for positive welfare such as living in a herd and accessing different types of enrichment (50, 54). These sorts of environments are becoming increasingly popular among horse owners regardless of equine body condition, and could lead more owners to manage weight incidentally rather than actively. Given that attitudes have been shown to follow behavior (14), this could lead to increased recognition and management of weight by such owners.

The complexity of equine weight management highlights the importance of tailoring weight management strategies to the individual human-horse dyad, in a similar manner to the tailoring recommended also in dog obesity management (49). Because owners found it difficult to implement changes which brought about negative experiences (hunger, frustration) in their horses, our findings also suggest that there would be merit in in encouraging owners to consider positive welfare during weight management. For example, if owners are encouraged to consider incorporating equine needs (for example, having companions while on restricted grazing, having different types of low-calorie forage to promote choice, having enrichment such as scratching posts, toys, and obstacles) this could not only optimize the horse's wellbeing but also help owners to manage their own emotions during weight management, and continue with regimen over time. To this end, the authors have created a weight management decision making tool, which helps owners reflect on the opportunities for change available to them and their horse as individuals, and promotes positive welfare (36).

This study has some limitations: qualitative research methods aim to create a deep understanding of participants' experience, rather than to create a generalizable sample of data. Therefore, the application of a categorical framework such as the COM-B model could be at odds with the data collected; real-life data are complex and, indeed, there were examples of data which did not neatly fit in one category or other (for example, owners' psychological capability to recognize and respond to overweight was often closely linked to their motivation to change). Secondly, all data were self-reported by owners and hence subject to biases, such as confirmation and hindsight bias. Future ethnographic work could observe the real day-to-day issues and experiences of horse owners when recognizing and managing weight.

## Conclusion

This study has revealed valuable information about the complexities of managing equine obesity, highlighting that weight management is about more than just “educating the owner” and expecting them to make successful changes. The application of the COM-B model highlights the multiple obesogenic influences on horse owners and the horses in their care, and thus reveals the reasons that equine obesity has become so prevalent.

Making changes to equine obesity prevalence will therefore require the implementation of interventions at multiple levels, increasing horse owner knowledge, motivation and social support. Additionally, creating non-obesogenic physical environments which fulfill horses' behavioral needs will assist owners in better managing their health and weight proactively. A collaborative approach which unites horse owners, veterinarians, welfare professionals, nutritionists, behaviorists, farriers and other equine professionals in tackling obesity proactively and at multiple levels would assist the creation of non-obesogenic environments and equine care practices.

## Data availability statement

The raw data supporting the conclusions of this article will be made available by the authors, without undue reservation.

## Ethics statement

The studies involving human participants were reviewed and approved by University of Liverpool Veterinary Ethics Committee. The patients/participants provided their written informed consent to participate in this study.

## Author contributions

Data collection and manuscript preparation: TF. Grounded theory data analysis: TF, EP, FW, and RC. COM-B analysis: TF and RC. Project conception and design and manuscript editing: All authors. All authors contributed to the article and approved the submitted version.

## Funding

The research resulting in this article was funded by a Ph.D. studentship funded by The Horse Trust.

## Conflict of interest

The authors declare that the research was conducted in the absence of any commercial or financial relationships that could be construed as a potential conflict of interest.

## Publisher's note

All claims expressed in this article are solely those of the authors and do not necessarily represent those of their affiliated organizations, or those of the publisher, the editors and the reviewers. Any product that may be evaluated in this article, or claim that may be made by its manufacturer, is not guaranteed or endorsed by the publisher.
